# Correction: The impact of rapid urbanization on residential energy consumption in China

**DOI:** 10.1371/journal.pone.0283035

**Published:** 2023-03-08

**Authors:** The PLOS ONE Staff

## Notice of Republication

This article was republished on December 22, 2022, to correct for errors in the Data Availability statement. The publisher apologizes for the errors. Please download this article again to view the correct version. The originally published, uncorrected article and the republished, corrected article are provided here for reference.

## Supporting information

S1 File Originally published, uncorrected article. (PDF)

S2 File Republished, corrected article. (PDF)

## References

[1] Wu W, lin Y (2022) The impact of rapid urbanization on residential energy consumption in China. PLoS ONE 17(7): e0270226. 10.1371/journal.pone.0270226 35901022 PMC9333300

