# Supplementary material for: Correction: The impact of rapid urbanization on residential energy consumption in China
Source: PLoS One. 2023 Mar 8;18(3):e0283035. doi: 10.1371/journal.pone.0283035 (PMC9994693; doi:10.1371/journal.pone.0283035)
Supplement: S1 File — (PDF) [file pone.0283035.s001.pdf]

PLOS ONE

RESEARCH ARTICLE

The impact of rapid urbanization on residential energy consumption in China

Weilong Wu[1], **Youna lin**[2]*

1 School of Film Television & Communication, Xiamen University of Technology, Xiamen, China, 2 School of Applied Economics, Central University of Finance and Economics, Beijing, China

* youna_lin@163.com

Check for updates

OPEN ACCESS

Citation: Wu W, lin Y (2022) The impact of rapid urbanization on residential energy consumption in China. PLoS ONE 17(7): e0270226. https://doi.org/10.1371/journal.pone.0270226

Editor: Jun Yang, Northeastern University (Shenyang China), CHINA

Received: January 11, 2022

Accepted: June 7, 2022

Published: July 28, 2022

Copyright: © 2022 Wu, -lin. This is an open access article distributed under the terms of the Creative Commons Attribution License, which permits unrestricted use, distribution, and reproduction in any medium, provided the original author and source are credited.

Data Availability Statement: ***PA @ ACCEPT: Please follow up with authors for data at accept*** The dataset required for the study has been uploaded to the system along with the manuscript and will be published for download if the manuscript is accepted.

Funding: The author(s) received no specific funding for this work.

Competing interests: The authors have declared that no competing interests exist.

Abstract

Due to the rapid progress of urbanization in China, the percentage of residential energy consumption out of total energy consumption has increased. This paper uses statistical data from 30 Chinese provinces (autonomous regions and municipalities) from 2000 to 2020 to analyze the impact of urbanization on residential energy consumption and construct an econometric model to test the mechanism. The empirical tests show that the consumption of direct energy (energy that exists in nature in its original form and has not been transformed) is positively U-shaped about the urbanization rate. Furthermore, the impact of economic development on direct and indirect energy consumption is significantly positive. In contrast, the effects of population agglomeration on immediate energy consumption are adverse, and the indirect energy consumption is positive.

I. Introduction

Since the 21st century, China's rapid economic and social development has led to increasingly significant constraints on China's economic and social development and has had increasingly severe impacts on the natural environment on which people rely for survival. Therefore, understanding the characteristics of China's regional energy consumption is a prerequisite for and key to coordinating regional energy consumption with balanced regional development.

On the one hand, as the final destination of energy consumption, household energy consumption is increasingly becoming an essential part of China's energy consumption and the implementation of energy conservation and emission reduction. On the other hand, the rapid development of China's economy in recent years has dramatically improved the living standards of both urban and rural residents, which has led to a rapid increase in energy consumption, especially with the rapid progression of urbanization, resulting in significant changes in the structure and patterns of energy consumption by urban and rural residents in different regions of China. For a long time, along with the rapid development of China's economy, the issue of energy will become more prominent for the sustainable development of China. Therefore, by revealing the characteristics and patterns of changes in household energy consumption in China, this paper can provide a more comprehensive analysis of the mechanisms by which

urbanization affects residential energy consumption and thus provide a reference basis for relevant departments when formulating policies.

However, due to the vast territory of China, the natural resource endowments, development patterns, and levels of development vary significantly from region to region. Energy consumption is inevitably affected by various biological and socioeconomic conditions, so it is essential to analyze the spatial and temporal characteristics of energy consumption in different provinces and regions in China and to understand the patterns of energy consumption in each area in order to formulate differentiated energy management policies according to local conditions and to promote sustainable economic and social development.

Several studies have shown that urbanization and energy consumption are closely related [1]. For example, according to estimates by China's national statistics department, a 1% increase in the urbanization rate increases total energy consumption by at least 60 million tons of standard coal, so if China were to reach the 80% urbanization level of developed countries, total energy consumption would increase by at least another 1,288.8 million tons of standard coal [2]. Therefore, as urbanization continues, the relative lack of energy will become a significant constraint on China's economic and social development [3]. How can urbanization and residential energy consumption be developed synergistically? This paper first compares the relevant theoretical and empirical studies on the relationship between urbanization and residential energy consumption; second, using data from 30 Chinese provinces (autonomous regions and municipalities) from 2000 to 2020, it constructs an econometric model for empirical testing based on the analysis of the mechanism of urbanization's impact on residential energy consumption. Finally, the conclusions drawn from these studies allow us to make policy recommendations.

To measure household energy consumption, this study mainly uses the China Family Panel Studies (CFPS) microdata. For this study, the following aspects of the survey data were used. First, the consumption expenditure items in the household questionnaire were used to measure the energy consumption of each household. In contrast, the household income and household size variables in the household questionnaire and the education level of the household head in the individual questionnaire were selected as the influencing factors of household energy consumption in the subsequent analysis. In addition, the sectoral energy consumption intensity data and input-output table data used in the subsequent measurement of household energy consumption were obtained from the World Input-Output Table Database WIOD. Decomposition weights and value conversion data were obtained from the input-output table of China's 135 sectors for urban and rural residents in each industry and annual CPI data, respectively, published by the National Bureau of Statistics. Finally, some of the influencing variables were obtained from previous years' statistical yearbooks.

The marginal contributions of this paper are as follows. First, most literature has focused on total energy consumption and the still large amount of industrial energy consumption. In contrast, little literature has focused on residential energy consumption. Since China entered its late industrialization phase, the proportion of residential energy consumption out of total energy consumption has increased each year. Therefore, this paper takes urbanization and residential energy consumption as the research object, thus filling the gap in the corresponding literature. It is therefore more relevant to reality, and the appropriate conclusions and policy recommendations have specific practical significance. Second, the theoretical value of this paper is that it provides a more comprehensive analysis of the mechanisms by which urbanization affects residential energy consumption from the perspectives of the scale effect, structural effect, and technology effect to more deeply understand the correlation between the two and provide a reference basis for the relevant departments when formulating policies. Third, most of the literature on residential energy consumption is based on the total energy consumption

of residents, ignoring the differences in the impact mechanisms of their internal institutions, i.e., direct and indirect energy consumption; thus, the conclusions obtained are rather general. This paper analyses residential energy consumption at three levels—direct, indirect, and complete—thus providing a comprehensive view.

II. Review of the literature

1. Theoretical studies on urbanization and energy consumption

The core elements of ecological modernization theory include technological innovation, market mechanisms, environmental policy, and the precautionary principle, with the market playing the first role [4, 5]. It can be summarized in four areas: first, the concept of compatible development. Technological innovation and institutional change can lead to consistent development between ecology and modernization in urban societies. Therefore, companies and governments should be more ecologically conscious and build a "climate and economy" approach to investment activities and policy formulation and implementation. "Therefore, companies and governments should be more ecologically conscious and build a two-way link between 'environment and economy' in the overal process of investment activities and policy formulation to implementation, but the public should also emphasize the awareness of environmental responsibility [6, 7]. The second is a systemic analysis tool. This is a tool for the systematic analysis of social development, bringing together various indicators—such as the degree of policy openness and green development—to analyze the key elements of socioeconomic development that determine ecological concepts and optimize industrial structures and to identify the intrinsic dynamics that drive green development in urban societies [5, 7]. Third is a practical platform. Ecological modernization theory is considered a suitable platform that can promote the green transformation of urban societies, advocating for the formation of a coordinated and cooperative government and enterprises to carry out this delicate operation [6, 8]. Fourth is market-led green change. In the process of urbanization, the market can solve energy and environmental problems more effectively than the government, and sustainable economic and social development can be driven by technological innovation and improvement and achieved along the path of urbanization [9, 10]. Ecological modernization theory suggests that low- and middle-income countries will have more energy and environmental problems. Technological innovation and increased environmental awareness may lead to a "decoupling" effect, the central development goal [9, 11, 12]. Thus, this theory considers the technological, institutional, and market drivers of the ecological transition in urbanization and is optimistic about subsequent environmental problems, energy consumption, etc. [13, 14].

Urban environmental transition theory suggests that the demand for energy from urban development and industrial production is phased. The efficiency of resource consumption is characterized by an inverted *U* shape of "low, high and low" [15]. The essence of urban transformation is to move from a traditional, energy-intensive, and crude model to a high-technology and ecological model. Therefore, different measures are needed at various stages of urban development to promote urban prosperity. According to the theory of urban environmental transformation, the administrative means to change urban development patterns include policy guidance, legal and regulatory constraints, and financial support, but market mechanisms are also important. Key milestones in the transition period for towns and cities include improving energy efficiency, developing and using renewable energy sources, and adopting low-carbon alternatives to the industry as a means of achieving innovation-driven green industry and green development of towns and cities [16, 17].

In its early days, compact town theory focused on the location and design of public transport hubs, the layout of functionally concentrated town centers, and self-sufficient community

distribution. Close towns can, to some extent, discourage excessive energy consumption due to town overgrowth, as they represent a higher town density and a more reasonable town size [18, 19]. Studies confirm that compact town development patterns can reduce energy consumption [20, 21]. However, if town infrastructure lags, continued urbanization will only lead to more severe energy and environmental problems. For example, a study found that Korea suffers from extremely inefficient congestion under a compact development model [22].

2. Empirical study of urbanization and energy consumption

By reviewing data for 59 developing countries in 1980, [23] found a positive relationship between urbanization and energy consumption. Urbanization benefits towns through economies of scale in production and increases transport and economical energy consumption. [24] modeled the relationship using country data and found that increased urbanization increases energy consumption per capita in three ways: first, by shifting the energy used from traditional to modern fuels; second, by increasing the demand for goods and services and so on; and third, by transport and household energy consumption. The findings suggest that due to China's backward energy consumption structure and rigid industrial structure evolution, increasing urbanization rates will increase energy consumption demand, especially in the central and western regions [25]. [26] developed an equilibrium equation representing the long-term trend of energy consumption in each area and found that the net effect of urbanization on energy consumption in China is positive, suggesting that the simple urbanization push has not fully exploited the agglomeration effect. [27] conducted an empirical analysis using the IPAT model and showed that the impact of urbanization on energy consumption in China is positive. The results of the empirical analysis using the IPAT model show that the effect of urbanization on energy consumption in China is positive, with an elasticity coefficient of 0.3. Based on this, we found that the positive impact is due to the significant increase in energy consumption in transport, consumption, and construction during urbanization. [28] constructed a two-class decomposition model, which shows that urbanization has contributed to the rise in direct energy consumption in China through changes in the population's lifestyle. Empirical analysis shows that urbanization and residential energy consumption are two-way Granger causes, with the former having a positive long-term effect on the latter[29]. [30] broke down residential energy consumption into six main products and showed that urbanization contributes more to this than population size. [31] used a GMM approach and a threshold regression model to construct transmission paths from direct and indirect effects, respectively, and tested the threshold effect. The results show that the expansion of urbanization and the improvement of quality of life in urbanization increase energy consumption. At the same time, the advanced industrial structure plays a contraction role. Nevertheless, its coefficient is relatively small at present, so overall, urbanization shows a gradual expansion trend that is diminishing. Studies on strategies to reduce energy consumption [32] concluded that improved ventilation could directly or indirectly reduce urban energy consumption. [33] used the log-average method to find that energy structure effects, economic effects, and population effects positively impact the growth of urban renewable energy consumption. [34, 35] systematically analyzed the historical and current situation of energy consumption in rural areas in recent years and proposed measures to reduce energy consumption in rural buildings based on strategies tailored to local conditions, including building planning and design, renewable energy development and utilization, and energy savings in daily life.

Most of the literature confirms a positive relationship between urbanization and energy consumption in empirical studies. One study indicates that the level of urbanization is significantly negatively related to the amount of fuel used [36]. [37] also found that regions with high

levels of urbanization in Canada had low per capita energy consumption. Using a questionnaire to compare residential energy consumption in India and China, rural residents consumed more energy than urban residents because inefficient solid fuels have long accounted for over 85% of residential consumers [38]. Other scholars have confirmed that the relationship between urbanization and energy consumption is phased. For example, [39] used the STIRPAT model to assess data for 73 countries and found that the relationship between urbanization and energy consumption is not the same for countries at different stages of economic development. Specifically, urbanization does not increase energy consumption for upper-middle-income and low-income countries, but the opposite is true for lower-middle-income countries. Thirteen Pacific Island countries, including Polynesia and Tonga, were studied from 1980–2005, and a negative relationship was found between urbanization and per capita energy consumption [40]. Eight regions of the world were studied, and a long-term positive association between urbanization and energy consumption was found in 84% of the countries. The remaining countries showed a negative association, and there was a non-significant association in some low-income countries [41].

3. The difference in energy consumption characteristics between urban and rural residents

Compared to rural areas, the types and quantities of energy supply are more abundant in towns and cities, and urban households have more energy-consuming appliances, such as air conditioners and heating equipment, and the per capita direct energy consumption of urban residents is generally higher than that of rural residents, and the numerical increase in urban population leads to a rise in total demand for residential energy consumption in society as a whole [42]. On the other hand, the massive shift of rural population to urban areas also affects residential direct energy consumption by influencing the production and consumption of agriculture. Because a significant reduction in rural population can lead to understaffing in agricultural work to meet the consumption needs of a larger population, large-scale mechanization of agriculture and the use of food transportation systems consume large amounts of fossil energy [43]. At the same time, the population size effect reduces the indirect energy consumption of the population. Because with the increase of population density, the rational planning of mass transportation such as metro facilitates the formation of a good spatial layout of towns, compact town development can reduce the supply and use costs of schools, water and electricity supply, public transportation, etc., and shorten the commuting distance, thus exerting a spatial agglomeration effect [21, 44, 45].

From the energy demand side, China's energy reserve structure is characterized by more coal, less oil and gas, which to a certain extent determines the structure of direct energy consumption by the population. Coal is a relatively inexpensive energy product, so people can only choose coal as the main energy consumption product when the residents' income is generally low. This can explain the difference in the structure of residential direct energy consumption between urban and rural areas, i.e., rural residents have a more homogeneous type of direct energy consumption, mainly consuming bioenergy and coal, such as animal manure and crop straw, while urban residents have a more diverse type of direct energy consumption and use a higher proportion of green energy such as natural gas and electricity. Therefore, with the increase of urbanization rate, more rural population turns into new urban population, and these new urban population, driven by the original urban population, also start to consume direct energy mainly by green energy, thus improving the structure of residential direct energy consumption and reducing residential direct energy consumption [46].

From the energy supply side, urbanization will promote the construction and improvement of regional infrastructure, which will facilitate the supply of green energy such as electricity and natural gas and improve the structure of residential direct energy consumption. Rural areas have relatively poor infrastructure, which makes it difficult to achieve a uniform supply of energy on a large scale, so it is usually done only on a small scale, for example, small villages use generators to supply electricity, and villages near water use river water or drill wells to obtain water. In contrast, cities and towns have relatively complete infrastructure, such as large power supply networks, water supply and drainage systems, and the supply of natural gas, etc. also relies on a large pipeline delivery system. Therefore, as urbanization progresses to rural areas, the supply of green energy such as electricity and natural gas in rural areas is greatly facilitated by the installation of larger and larger energy supply networks and pipelines [47]. As the proportion of green energy in the direct residential energy supply increases, the structure of the energy supply side will continue to improve, which further promotes the improvement of the direct residential energy consumption structure, i.e., rural residents will also gradually reduce their consumption of coal and choose to consume green energy such as electricity and natural gas, thus reducing the direct residential energy consumption [38]. However, indirect residential energy consumption increases during the period when the initial energy networks and systems are being built in large quantities. Therefore, the impact of structural effects on the total residential energy consumption varies with the stage of urbanization.

4. Summary

To more clearly summarize the literature on the relationship between urbanization and energy consumption, this paper has been categorized and summarized as shown in Table 1 below.

The theories can be divided into ecological modernization theory, urban environmental transition theory, and compact town theory at the theoretical level. In addition, there is a large body of literature domestically and abroad on the practical level, using various econometric models for regression analysis. In general, the empirical results of the impact of urbanization on energy consumption can be divided into three main categories: positive, negative, and non-linear relationships. Most of the literature focuses on total energy consumption and industrial energy consumption, which account for the largest share of energy consumption. In contrast, very little of the literature focuses on residential energy consumption. Most of this lesser literature uses residential direct energy consumption as a proxy for residential energy consumption and does not classify residential energy consumption. This is not comprehensive. According

Table 1. Summary of the literature.

Theoretical Research	Ecological Modernisation Theory	The concept of compatible development	(Baris, 2011; Stringer et al., 2014)
		Systematic analysis tools	(Buttel, 2000; Stringer et al., 2014)
		Practical platform	(Baris, 2011; Mol & Arthur, 2006)
		Market-led green change	(Krishnamurthy & KristrM, 2016; V. L. Smith & Font, 2014)
	Urban Environmental Transformation Theory	(Christoff, 2007; Lynn & Kevin, 2013; Musakwa & Niekerk, 2013)	
	Compact Town Theory	(Czamanski & Roth, 2011; Keisuke et al., 2018; Lee & Lee, 2014; Shammin et al., 2010)	
Empirical Studies	Positive Correlation	(Fang et al., 2015; Jiang & Lin, 2013; Jones, 2007; Parikh & Shukla, 1995)	
	Negative correlation	(Ke & Lin, 2015; Lafrance, 1999; Pachauri & Jiang, 2008; Pucher, 1989)	
Study of strategies to reduce energy consumption	(He, Li, et al., 2014; He, Yang, et al., 2014; J Yang et al., 2016; Jun Yang et al., 2021)		

https://doi.org/10.1371/journal.pone.0270226.t001

to this paper, immediate residential energy consumption accounts for less than one-third of residential energy consumption. The mechanisms that influence direct and indirect residential energy consumption are not identical and cannot be generalized.

III. Methodology

1. Variable selection and data sources

This paper uses panel data from 30 Chinese provinces (regions and municipalities, excluding Tibet) from 2000 to 2020. The variables involved can be divided into three categories, and Table 2 below shows the meaning of each variable and the descriptive statistical analysis.

The first category is the explanatory variables: direct, indirect, and complete energy consumption of the population. Immediate household energy consumption includes the direct purchase and use of different types of energy; such as electricity, coal, and natural gas; by residential households or the production sector. For the production sector, direct energy consumption uses end-use energy such as coal and electricity in the production process. The household energy consumption studied in this paper mainly refers to the energy demand for household lighting, heating, water use, transport, and travel behavior. Household indirect energy consumption, including all non-direct energy goods and services purchased for the needs of residents' household life, such as food, clothing, culture and entertainment, medical education, etc. The energy consumed to produce, process, transport, sell and dispose of these goods and services is indirect household energy consumption. Particular attention should be given to the consumption of service goods. Before these non-energy goods and services reach the consumer, their processes are bound to generate a certain amount of energy consumption, such as the consumption of education, which also indirectly consumes energy, as the purchase of these goods and services inevitably leads to the consumption of energy by other related industries, such as the paper industry and the printing industry. Hence, household energy consumption is not simply a matter of ours. Therefore, it is not merely the direct energy consumption of electricity and fuel that we intuitively understand but which is also closely related to our lives.

Moreover, direct energy consumption and indirect energy consumption are not opposed to each other but do have different meanings. For example, in the case of thermal power plants, the purchase of coal for power generation is direct energy consumption, while for end consumers, the purchase of various goods and services to meet their needs indirectly brings about the consumption of electricity, which is indirect energy consumption. Therefore, for

Table 2. Model-related variable settings and descriptive statistical analysis.

	Variable Meaning	Symbol	Mean value	Standard deviation	Min	Maximum value
Explained variables	Direct residential energy consumption (kg of standard coal/person)	perDe	6.8897	0.5469	5.242	753.832
	Indirect energy consumption of the population (kg of standard coal/ person)	perIe	5.609	0.3583	3.412	7.102
	Total energy consumption of the population (kg of standard coal/ person)	perTe	6.5256	0.6217	4.882	733.832
Explanatory variables	Urbanisation rate (%)	Urb	4.4317	0.3274	3.2905	5.3274
Control variables	Degree of population agglomeration (persons/km2)	Pop	15.5385	2.3479	10.0548	21.0402
	Level of economic development (yuan)	perGdp	10.28	0.7358	8.082	12.192
	Average temperature in January (K)	Temp1	5.9842	1.2875	2.1121	9.088
	Average temperature in July (K)	Temp7	6.1859	0.0607	5.662	6.512

https://doi.org/10.1371/journal.pone.0270226.t002

residential households, indirect household energy consumption should be the consumption of non-energy goods and services in addition to the abovementioned inter-household energy consumption. Thus, total residential energy consumption equals the sum of direct and indirect residential energy consumption.

(1) The data on the direct energy consumption by residents were obtained from the China Energy Statistical Yearbook. The energy balance sheets (physical quantities) of each province (region and city) were converted into standard quantities according to the standard quantity conversion coefficients and summed to obtain residents' respective direct energy consumption. (2) The indirect energy consumption of residents was measured using the CLA method, in which data on residents' expenditure on various consumable items were obtained from the China Statistical Yearbook. In contrast, the industry's energy consumption and output value were obtained from the China Energy Statistical Yearbook and the China Industrial Statistical Yearbook, respectively. At the same time, to exclude the influence of price changes, this paper uses the year 2000 as the base period for converting the uniform price index. (3) The total energy consumption of residents equals the sum of residents' direct and indirect energy consumption.

The second category is the key explanatory variable, namely, the urbanization rate. Although new urbanization has a rich connotation, the core meaning is people-oriented, urban-rural integration, ecological livability, and coordinated and sustainable development. Therefore, to avoid the possible bias brought by using a single indicator of the population urbanization rate to measure the new urbanization level, this paper establishes a comprehensive evaluation index system of 16 indicators of the new urbanization level in four dimensions: population, economy, society, and environment, as shown in Table 3. Furthermore, the specific indicators are relative to per capita and proportion to enhance the spatial and temporal comparability of collaborative development. The original data were mainly obtained from the China Statistical Yearbook, China Rural Statistical Yearbook, China Health and Health Statistical Yearbook, China Urban Construction Statistical Yearbook, and the statistical yearbooks of various provinces from 2000 to 2020, with missing data obtained by interpolation. Finally, this paper applies the entropy weighting method to determine the index weights of the

Table 3. Comprehensive evaluation index system of the new urbanization level.

Total indicators	Secondary indicators	Description of the hand (in units)
V1 Population urbanisation	Share of non-farm workers	Number of employees in secondary and tertiary sectors/employees at the end of the year (%)
	Urban population density	Urban population/urban area (persons/km2)
	Urban population employment	Registered urban unemployment rate (%)
V2 Economic urbanisation	Level of economic development	Gross regional product per capita (yuan)
	Economic structure	Output value of non-agricultural industries/regional GDP (%)
	Investment in science and technology	Share of expenditure on science and technology in fiscal spending (%)
V3 Social urbanisation	Financial level	Local general budget revenue per capita (yuan)
	Education level	Share of expenditure on education in fiscal spending (%)
	Medical level	Number of hospital and health center beds per 1,000 population (beds per person)
	Public Cultural Services	Number of public libraries per capita (books)
	Public Transport	Public transport vehicles per 10,000 population (vehicles)
	Infrastructure Development	Urban road area per capita (sq m)
V4 Environmental Urbanisation	Parks and Green Spaces	Green space per capita (sq m)
	Urban Greening	Greening coverage of built-up areas (%)
	Exhaust Gas Emission	Industrial sulfur dioxide emissions per capita (tonnes)
	Sewage Discharge	Industrial effluent discharge per capita (million tonnes)

https://doi.org/10.1371/journal.pone.0270226.t003

evaluation system for the coordinated development of new urbanization and rural revitalization. It derives the new urbanization rates of 30 provinces in China from 2000 to 2020 through a multivariate comprehensive evaluation method.

The third category is the control variables, including (1) the degree of population accumulation, measured by the population density of the region, with data from the China Statistical Yearbook; (2) the level of economic development, measured by GDP per capita, with data from the China Statistical Yearbook and deflated using 2000 as the base period; and (3) the average temperature of each province in January and July, measured by the capital city of each province's January and July. The average temperature in January and July for each province is replaced by the average temperature in January and July in the capital city of each province, respectively, with data obtained from the China Statistical Yearbook.

2. Model construction

In this paper, we refer to the STIRPAT model proposed by Dietz and Rosa (1994).

$$I_{it} = aP_{it}^{b}A_{it}^{c}T_{it}^{d}e_{it} \tag{1}$$

where I, P, A, and T represent environmental impact, population size, level of economic development, and technological progress, respectively; a is a constant term; b, c, and d represent the elasticities of population size, level of economic growth, and technological progress on environmental impact, respectively; and e is a random disturbance term. After the equation is taken logarithmically twice at the same time, the model is transformed into

$$\ln I_{it} = \ln a + b\ln P_{it} + c\ln A_{it} + d\ln T_{it} + e_{it} \tag{2}$$

In this paper, the STIRPAT model described above is adapted accordingly to the purpose of our study, and the urbanization rate is introduced into the STIRPAT model as a proxy variable for T. Since residential energy consumption is taken as the mean value of people, population density is used instead of population size. Thus, Eq (2) can be expressed as

$$\ln E_{it} = \ln a + b\ln perGdp_{it} + c\ln Pop_{it} + d\ln Urb_{it} + e_{it} \tag{3}$$

Eq (3) is a standard linear model. At the same time, according to the analysis of the impact mechanism of urbanization on residential energy consumption in Chapter 4, it can be concluded that urbanization mainly affects residential energy consumption through the scale effect, structural effect, and technology. There is an interrelationship between different driving factors. Ultimately, the impact of urbanization on residential energy consumption is subject to multiple outcomes, and there may be a nonlinear relationship. Therefore, to further analyze whether there is a nonlinear relationship between urbanization and residential energy consumption, this paper introduces a quadratic term of the urbanization rate into Eq (3). Thus, Eq (3) can be expressed as

$$\ln E_{it} = \alpha_i + \beta\ln perGdp_{it} + \gamma\ln Urb_{it} + \gamma'\ln^2 Urb_{it} + \delta\ln Pop_{it} + e_{it} \tag{4}$$

The residential energy consumption studied in this paper is made up of both residential direct energy consumption and residential indirect energy consumption, but as the analysis of the current situation in the previous report concludes, the proportion of residential indirect energy consumption reaches more than 2/3, and the balance of residential direct energy consumption is much smaller if the two are added together. Therefore, only the regression of residential complete energy consumption is considered. The residential immediate energy consumption characteristics will be neglected, so this paper also analyzes the population's

direct and indirect energy consumption separately. Furthermore, considering that urbanization also has a somewhat different mechanism of influence on these two types of energy consumption, with heating and cooling accounting for a more significant proportion of residential direct energy consumption, this paper adds the average temperature in January and the average temperature in July as control variables in the econometric model of residential immediate energy consumption. As a result, the model for residential direct, indirect, and total energy consumption is built.

$$\ln perDe_{it} = \alpha_i + \beta \,\mathrm{lnper}\, Gdp_{it} + \gamma \ln Urb_{it} + \gamma' \ln^2 Urb_{it} + \delta \ln Pop_{it} + \mu_1 \ln Temp1_{it} + \mu_2 \ln Temp7_{it} + e_{it} \quad (5)$$

$$\ln perIe_{it} = \alpha_i + \beta \ln perGdp_{it} + \gamma \ln Urb_{it} + \gamma' \ln^2 Urb_{it} + \delta \ln Pop_{it} + e_{it} \quad (6)$$

$$\ln perTe_{it} = \alpha_i + \beta \ln perGdp_{it} + \gamma \ln Urb_{it} + \gamma' \ln^2 Urb_{it} + \delta \ln Pop_{it} + \mu_1 \ln \mathrm{Temp1}_{it} + \mu_2 \ln \mathrm{Temp\ 7}_{it} + e_{it} \quad (7)$$

IV. Results

1. Baseline regression results

(1) The non-linear relationship between urbanization and residential energy consumption in China. As we can see from Table 4 and Fig 1, regarding the key explanatory variable of the urbanization rate, in China, the coefficient of larb is -8.449. The coefficient of $(\mathrm{lnUrb})^2$ is 1.256. Both are significant at the 1% level of significance, which indicates that there is a positive U-shaped relationship between the impact of urbanization on residential direct energy

Table 4. Estimation results of the residential direct energy consumption model.

Variables	30 provinces nationwide	11 eastern provinces	8 Central Provinces	11 western provinces
	Fixed effects model	Random effects model	Fixed effects model	Fixed effects model
lnUrb	-8.449***	-7.045***	-6.348**	-7.159**
	(-5.25)	(-3.48)	(-2.41)	(-2.53)
$(\mathrm{lnUrb})^2$	1.256***	0.971**	0.924**	0.960**
	(-5.515)	(-3.145)	(-2.147)	(-2.1415)
lnperGdp	0.545***	0.515**	0.474***	0.441**
	(-9.015)	(-10.552)	(-6.522)	(-2.974)
lnPop	-0.274	-0.152	-2.374**	-2.010***
	(-1.15)	(-1.26)	(-3.74)	(-4.262)
lnTemp1	-1.115	4.74**	1.187	0.91
	(-0.74)	(-2.86)	(-0.674)	(-0.522)
lnTemp7	-0.157	1.357	0.915	-2.374
	(-0.26)	(-0.488)	(-0.345)	(-0.15)
Constants	27.752	27.845	12.215	30.610
	-1.802	-1.508	-0.522	-1.082
R^2	0.6909	0.8315	0.774	0.5441
Sample	556	125	148	192

Note: Data in brackets in the table are t-statistics;

***, ** and * indicate significance at 1%, 5% and 10%, respectively.

In this paper, the eastern region includes 11 provinces, including Beijing, Tianjin, Hebei, Shanghai, Jiangsu, Zhejiang, Fujian, Shandong, Guangdong, Liaoning and Hainan, according to the division standard of the National Bureau of Statistics of China. The central region includes eight provinces of Shanxi, Anhui, Jiangxi, Henan, Hubei, Jilin, Heilongjiang and Hunan. The western region includes Inner Mongolia, Guangxi, Chongqing, Sichuan, Guizhou, Yunnan, Shaanxi, Gansu, Qinghai, Ningxia and Xinjiang 11 provinces.

https://doi.org/10.1371/journal.pone.0270226.t004

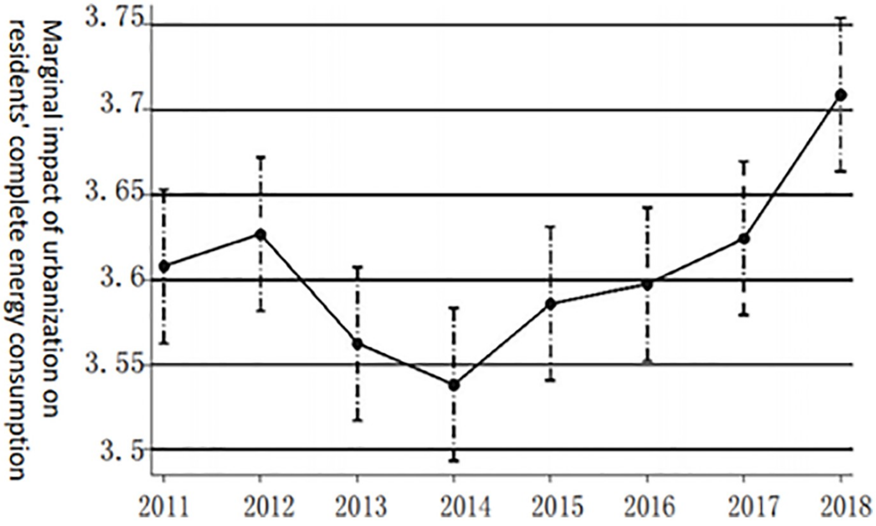

Fig 1. Dynamic effect of urbanization on the marginal impact of complete energy consumption of the population.

https://doi.org/10.1371/journal.pone.0270226.g001

consumption in China. For example, before the turning point, as the urbanization process in China progresses, the residential immediate energy consumption decreases, while when urbanization reaches a certain level after the turning point, residential direct energy consumption increases as the urbanization rate rises further. Similar to the model for total residential energy consumption, based on these two coefficients, we can conclude that the urbanization rate corresponding to the turning point of residential direct energy consumption is 40.13%. As of 2017, China's urbanization rate has reached 58.52%, so China has entered the right-hand side of the positive U-shaped curve since 2003, with a positive correlation between residential direct energy consumption and the urbanization rate.

(2) Regional heterogeneity. The urbanization rate and its quadratic term are significant at the regional level in the East, Central and West China models. Based on their coefficients, it can be seen that there are also positive U-shaped relationships between the urbanization rate and residential direct energy consumption in East, Central, and West China, with inflection point values of 36.94%, 31.39%, and 38.90%, respectively. Currently, residential direct energy consumption in all three regions is on the right side of the positive U-shaped curve. Apart from the key explanatory variable of the urbanization rate, the coefficient on GDP per capita is positively significant at both the Chinese and regional levels, with residential direct energy consumption increasing by approximately 0.5% with a 1% increase. On the other hand, the coefficient on population density is negative at both the national and sub-regional levels, suggesting that population agglomeration favors lower residential direct energy consumption. The possible reason for this is that as population density increases, the rational planning of mass transport modes such as metro and light rail, as well as the more centralized provision of infrastructures such as heating and gas, facilitates the formation of a good spatial layout in towns and cities, saving intermediate costs in transport and living production and bringing into play the spatial agglomeration effect, thus reducing residential direct energy consumption to a certain extent. It can also be shown that the positive impact of population agglomeration is better for the central and western regions with lower population density than for the eastern areas with higher population density. In addition, the average temperature in January has a more pronounced effect on energy consumption in the eastern regions, with a negative correlation with direct residential energy consumption; the lower the average temperature is, the

more energy is consumed. This may be because heating and cooling equipment is more widespread in the eastern regions. Therefore, residential energy consumption changes more significantly with temperature changes. In contrast, due to the lower prevalence of such equipment in the central and western areas, residential energy consumption is less affected by temperature changes.

2. Extended results: An indirect energy consumption perspective

By regressing the model with the quadratic term of urbanization rate included, we found that the urbanization rate and most of the control variables were not significant, probably because the model for residential indirect energy consumption was more in line with the linear relationship. Hence, the paper was re-run with the quadratic term of urbanization rate removed, and Table 5 shows the estimated results of the linear regression. At the Chinese level, the coefficient of larb is 0.194, which indicates that for every 1% increase in the urbanization rate, residential indirect energy consumption increases by 0.194%, which shows a growing residential indirect energy consumption trend with urbanization. This is mainly because economic scale effects strongly influence residential indirect energy consumption. Urbanization increases the income of the population, which, according to the traditional theory of consumer demand, stimulates the people's consumption demand, thus leading to an increase in indirect energy consumption. From the model results, it is clear that the current change in residents' consumption attitudes, such as the awareness of green consumption, has not yet played a more significant role.

3. Extended results: A complete energy consumption perspective

(1) Non-linear relationship. From Table 6 we can see that, regarding the key explanatory variable of urbanization rate, at the Chinese level, the coefficient of larb is -3.3658. Likewise, the coefficient of (lnUrb)2 is 0.494. Both are significant at the 1% level of significance, which indicates that there is also a positive U-shaped relationship between the impact of urbanization on residential complete energy consumption in China, and, based on these two coefficients, we can conclude that The urbanization rate corresponding to the inflection point of residential entire energy consumption is 36.24%. As of 2017, China's urbanization rate has reached

Table 5. Estimation results of the residential indirect energy consumption model.

Variables	30 provinces nationwide	11 eastern provinces	8 Central Provinces	11 western provinces
	Fixed effects model	Fixed effects model	Random effects model	Random effects model
lnUrb	0.194***	-0.015	0.349***	0.048
	(3.16)	(8.02)	(6.52)	(0.02)
lnperGdp	0.774***	0.815***	0.702**	0.852
	(52.15)	(23.45)	(41.52)	(33.52)
lnPop	0.674***	0.523*	0.715**	0.641**
	(12.17)	(6.15)	(2.02)	(5.521)
Constants	-5.155***	-6.174**	-3.652**	-2.925*
	(-21.58)	(-9.52)	(-8.52)	(-21.02)
R^2	0.9845	0.9715	0.9893	0.995122
Sample	510	187	136	187

Note: Data in brackets in the table are t-statistics;

***, ** and * indicate significant at 1%, 5% and 10% respectively.

https://doi.org/10.1371/journal.pone.0270226.t005

Table 6. Estimation results of the residential complete energy consumption model.

Variables	30 provinces nationwide	11 eastern provinces	8 Central Provinces	11 western provinces
	Fixed effects model	Fixed effects model	Fixed effects model	Fixed effects model
lnUrb	-3.658***	-1.752**	-0.415	-5.015**
	(-5.15)	(-1.01)	(-0.05)	(-0.52)
$(\ln Urb)^2$	0.494***	0.226	0.152	0.715**
	(4.26)	(1.03)	(0.32)	(3.15)
lnperGdp	0.752**	0.815**	0.6023**	0.5526**
	(25.69)	(20.023)	(24.524)	(7.052)
lnPop	0.315**	0.352**	-0.6152**	-0.915**
	(4.52)	(4.52)	(-2.12)	(-3.02)
lnTemp1	-0.852	-2.156**	-0.002	0.352
	(-1.61)	(-3.063)	(-0.09)	(0.02)
lnTemp7	-0.552	-0.352	0.415	-1.215
	(-0.55)	(-0.02)	(0.41)	(-0.66)
Constants	11.545**	13.002	1.302	-8.552
	(1.62)	(1.58)	(0.16)	(1.36)
R^2	0.9552	0.9787	0.9715	0.9251
Sample	5152	102	126	155

Note: Data in brackets in the table are t-statistics;

***, ** and * indicate significant at 1%, 5% and 10% respectively.

https://doi.org/10.1371/journal.pone.0270226.t006

58.52%, so China's residential complete energy consumption is also currently on the right side of a positive U-shaped curve.

(2) Regional heterogeneity. At the regional level, the coefficients on the urbanization rate are significant in the models for 11 eastern provinces and 11 western provinces, except for the model for 8 central areas. The urbanization rates in China's eastern and western regions were 66.05% and 49.59%, respectively, in 2016, the same as at the Chinese level, and both are already on the right side of the positive U-shaped curve. For the eight central provinces, the urbanization rate does not significantly impact the total energy consumption of the population, but rather the level of economic development and population agglomeration play a more significant role.

4. Robustness test

To verify the robustness of the above model, this paper uses the Generalised Method of Moments Estimation (GMM) method to test it, treating the primary term of the urbanization rate as an endogenous variable and taking its lagged period as the instrumental variable. According to the results in Tables 7–9, we can see that the coefficient signs of the core explanatory variables and other control variables do not change in the model, except for individual variables that were not significant in the original model, so the previous model is more reasonable, and there is no problem of pseudo-regression.

V. Discussion

1. Discussion of regression results

As the analysis of the influence mechanism points out, the left side of the positive U-shaped curve of residential direct energy consumption occurs mainly due to the structural and qualitative effects. In contrast, the right side occurs mainly due to the economic scale and rebound

Table 7. GMM estimation results of the residential direct energy consumption model.

Variables	30 provinces nationwide	11 eastern provinces	8 Central Provinces	11 western provinces
lnUrb	-16.141***	-26.315***	-11.345	-2.915
	(-7.43)	(-6.42)	(-1.45)	(-0.33)
$(\ln Urb)^2$	2.002**	3.352***	1.656	0.445
	-7.712	-6.345	-1.504	-0.359
lnperGdp	0.5102***	0.653***	0.252*	0.191*
	-8.526	-8.456	-1.952	-1.694
lnPop	-0.525***	-0.356**	0.188**	0.003
	(-6.5)	(-4.5)	-1.974	-1.214
lnTemp1	-2.236**	-5.363***	1.617	-0.611**
	(-3.62)	(-5.37)	-1.064	-1.404
lnTemp7	-4.752**	12.46**	-14.049**	0.155***
	(-2.26)	-3.103	(-3.49)	(-5.26)
Constants	11.27	10.23	91.795**	130.238*
	-6.505	-0.318	-4.9023	-5.704
R^2	0.902	0.972	0.5903	0.523

Note: Data in brackets in the table are t-statistics;

***, ** and * indicate significant at 1%, 5% and 10% respectively.

https://doi.org/10.1371/journal.pone.0270226.t007

effects. At low levels of urbanization, the structural impact is that on the energy demand side, more rural people turn to new towns and cities, and driven by the former urban population, direct energy consumption tends to be dominated by renewable energy sources, thus improving the structure of immediate energy consumption by the people and reducing it. Conversely, on the energy supply side, urbanization leads to an increasing range of energy supply networks and pipelines being laid in rural areas. As a result, the share of renewable energy sources such as electricity and natural gas in the direct energy supply of the population is increasing, further improving the structure of people's immediate energy consumption and reducing their direct energy consumption. The qualitative effect is that urbanization brings about a concentration of people, resources, and knowledge and promotes more efficient energy use, higher productivity, and greater technological progress. New technologies can change the process of energy exploration and development and increase the development and use of new and clean energy

Table 8. GMM estimation results of the residential indirect energy consumption model.

Variables	30 provinces nationwide	11 eastern provinces	8 Central Provinces	11 western provinces
lnUrb	0.052	0.4526**	0.452**	0.26**
	(0.22)	(3.26)	(3.61)	(3.6)
lnperGdp	0.752**	0.823**	0.626**	0.726**
	(25.36)	(16.52)	(15.52)	(33.52)
lnPop	0.019**	-0.052	0.152**	0.152
	(2.05)	(-0.34)	(6.25)	(14.52)
Constants	-1.51**	-3.552**	-3.315**	-2.752**
	(-12.52)	(-15.51)	(-7.63)	(-25.52)
R^2	0.875	0.8652	0.901	0.9652

Note: Data in brackets in the table are t-statistics;

***, ** and * indicate significant at 1%, 5% and 10% respectively.

https://doi.org/10.1371/journal.pone.0270226.t008

Table 9. GMM estimation results of the residential complete energy consumption model.

Variables	30 provinces nationwide	11 eastern provinces	8 Central Provinces	11 western provinces
lnUrb	-7.952***	-10.452***	-3.8526	-3.626
	(-6.26)	(-3.26)	(-0.45)	(-0.52)
$(\ln Urb)^2$	1.026***	1.56***	0.626	0.452
	(6.23)	(4.26)	(0.26)	(0.52)
lnperGdp	0.626***	0.265***	0.563**	0.463
	(19.029)	(17.063)	(8.36)	(8.032)
lnPop	-0.126***	-0.126***	0.026	0.06**
	(7.26)	(-3.65)	(1.26)	(2.03)
lnTemp1	2.012***	0.726	2.926**	2.502
	(4.69)	(1.16)	(4.26)	(-0.26)
lnTemp7	-1.052	9.952***	-3.352	-8.023**
	(-0.15)	(4.25)	(-1.65)	(-5.52)
Constants	9.726	-41.82***	9.252	42.626
	(1.56)	(-3.52)	(0.85)	(4.29)
R^2	0.842	0.882	0.822	0.836

Note: Data in brackets in the table are t-statistics;

***, ** and * indicate significant at 1%, 5% and 10% respectively.

https://doi.org/10.1371/journal.pone.0270226.t009

sources, thus reducing direct energy consumption by the population. However, when the level of urbanization rises continuously, factors such as the production and transport of agriculture, the negative externalities of urban living, and the rebound effect of the increased propensity of residents to consume energy will lead to a renewed rise in indirect energy consumption by the population. In particular, the high consumption-driven lifestyle of the postindustrial period has been proven in some developed cities in Europe and the U.S. However, the positive effects of technological advances in new energy sources and increased awareness of energy efficiency among the population have not yet been effective in controlling this rebound effect.

At the regional level, indirect energy consumption in the eight central provinces is more significantly influenced by urbanization. In terms of the coefficient, for every 1 percentage point increase in the urbanization rate, the indirect energy consumption of residents in the central region increases by 0.396 percentage points. The coefficient for the 11 eastern provinces is negative but not significant; the coefficient for the 11 western provinces is similarly insignificant. The indirect energy consumption of residents in these two regions is more significantly influenced by economic development and population density. Apart from the key explanatory variable of urbanization rate, the coefficient on GDP per capita is positive and significant at both the Chinese and regional levels, with residential indirect energy consumption increasing by between 0.7% and 0.9% for every 1% increase. The coefficients for population density are also significant. Still, in contrast to the direct residential energy consumption results, population agglomeration has a more pronounced effect on indirect residential energy consumption. This effect decreases in the east, central and western regions, which means that to control the growth of indirect residential energy consumption, the population should be less concentrated in the densely populated eastern areas and more concentrated in the less densely populated central and western regions. To control the growth of indirect energy consumption, the population should be concentrated less in the densely populated eastern areas and more in the less densely populated central and western regions.

This paper proposes the following suggestions on how to guide the return of the employed population from the eastern region to the central and western areas. First, small and medium-sized towns in the west and central areas should make full use of the strategic advantage of taking over the industrial transfer from the eastern coastal areas to develop local industries and tertiary industries to enhance the ability to absorb labor force and to provide jobs for the returning migrant workers to small and medium-sized towns, as well as to fundamentally solve the problem of labor force left at home due to unemployment. Secondly, small and medium-sized cities in central and western regions should continuously improve the education system, increase investment in education, improve the quality of education, and focus on solving the situation that children of the migrant population stay at home or go to other regions due to the high cost of education facilities or education and the low quality of local education. Finally, to solve the problems of medical insurance and health care for the elderly left behind by migrant families, small and medium-sized towns in the central and western regions should step up efforts to improve the local medical insurance system, focus on implementing policies that favor medical insurance for the elderly left behind, and enhance the health care level of local medical institutions by introducing advanced technologies to fundamentally solve the medical insurance and health problems of the elderly left-back. Through the above means, we will gradually enhance the ability of small and medium-sized towns in the outflow areas to absorb the returning population and drive the majority of the floating population to settle in small and medium-sized cities in the outflow areas.

The reason for the positive U-shape of the complete residential energy consumption curve maybe because, in the early part of the study period, direct residential energy consumption accounted for a more significant proportion of the total energy consumption, and therefore in this period, total residential energy consumption decreased due to the decrease in direct residential energy consumption. However, in the latter part of the study period, indirect residential energy consumption increases faster after crossing the inflection point. As a result, it becomes a much more significant proportion of total residential energy consumption. Thus, total residential energy consumption increased due to the increase in indirect residential energy consumption.

In addition to the key explanatory variable of the urbanization rate, the other control variables' coefficients are also of interest. The coefficient on GDP per capita is positively significant at both the Chinese and regional levels, with residential complete energy consumption increasing by between 0.55% and 0.85% for every 1% increase. The coefficients for population density are also significant, but the sign of the coefficients varies between regions. For example, the coefficient on population density is positive for China and the eastern part, indicating that population agglomeration increases residential complete energy consumption. In contrast, the opposite is true for the central and western regions. This may be because the scale effect of population agglomeration has a critical point. When the degree of aggregation crosses the crucial moment, the positive impact of the scale effect will change to a negative one. A moderate population concentration is beneficial to reducing energy consumption, while an excessive population concentration is detrimental to reducing energy consumption. In addition, the effect of temperature is similar to the results in the model for direct residential energy consumption.

2. Recommendations

Based on the above key findings, this paper attempts to make the following policy recommendations for the synergistic development of China's urbanization and residential energy consumption.

1. Since total residential energy consumption encompasses both direct and indirect residential energy consumption, the influencing factors of both should be taken into account in the formulation of public policies. On the one hand, the scale and speed of infrastructure construction and the improvement of energy technology efficiency should be systematically planned and managed. On the other hand, residents' lifestyle consumption should be guided appropriately to enhance their awareness of energy conservation.
2. To effectively control the rebound effect of urbanization, clean energy development and the research and development of new energy-saving technologies should continue to be stepped up to improve energy efficiency. At the same time, frugal living should be promoted to prevent a surge in energy consumption after high urbanization.
3. Population agglomeration can, on the one hand, bring into play the agglomeration effect of space, technology, and talent, reducing residents' energy consumption. Nevertheless, on the other hand, it places higher demands on energy-intensive sectors such as the transport industry, thus increasing residents' energy consumption. This paper makes it clear that moderate population agglomeration is conducive to reducing residential complete energy consumption. In contrast, excessive population agglomeration will increase residential energy consumption. Therefore, China should now focus more on promoting the development of small and medium-sized towns rather than mega towns and introduce policies to encourage more talent to move to the central and western regions to effectively reduce overall energy consumption levels.
4. There is a significant positive correlation between GDP per capita and residential energy consumption. When resources are scarce and the pressure on the environment increases, it is inevitable that China's economic development enters a new normal by not being "GDP-only" (Focus on high GDP growth regardless of environmental pollution and energy over-consumption). To solve the many deep-rooted conflicts accumulated in rapid development, China's GDP growth rate needs to be appropriately reduced to leave room for resources and the environment.

VI. Conclusions

This paper uses statistical data from 30 Chinese provinces (autonomous regions and municipalities) from 2000 to 2020 to measure disaggregate residential energy consumption, analyze urbanization's impact mechanism on residential energy consumption, and construct an econometric model for empirical testing. The main findings of the study are as follows.

1. Based on the descriptive statistical analysis of the categorical measurement data, it can be seen that the per capita consumption of urban residents in China is greater than that of rural residents in terms of both direct and indirect energy consumption; however, the per capita immediate energy consumption of urban residents tends to rise and then fall, while the per capita direct energy consumption of rural residents tends to increase; the per capita indirect energy consumption of both urban and rural residents tends to rise, and the per capita indirect energy consumption of urban residents increases. As a result, both urban and rural residents per capita indirect energy consumption is rising. Urbanization mainly impacts residential energy consumption through scale, structure, and technology. The scale effect has positive and negative impacts on residents' direct and indirect energy consumption through population size and economic hierarchy. The structural implications reduce the immediate energy consumption of the population but increase the indirect energy consumption of the people. The qualitative effect of technology reduces residential energy consumption, but the

rebound effect increases residential energy consumption. Because of the interconnected and mutually reinforcing relationship between the different drivers, there are various possible scenarios for the impact of urbanization on residential energy consumption.

2. Based on the estimation results of the econometric model, overall, the impact of urbanization on residential direct energy consumption and residential complete energy consumption in China is phased and has a positive U-shaped relationship. The left-hand side of the positive U-shaped curve for residential immediate energy consumption is mainly due to the structural and quality effects, while the right-hand side is primarily due to the economic scale and rebound effects. The positive U-shaped relationship for residential complete energy consumption may be because the share of residential direct energy consumption is relatively large in the early part of the study period. Therefore, direct residential energy consumption decreases due to residential energy consumption. In the later part of the study period, indirect residential energy consumption increases more quickly after the inflection point is crossed. As a result, it becomes a much more significant proportion of total residential energy consumption. Thus, residential complete energy consumption increases during this period due to the increase in residential indirect energy consumption. The urbanization rate in China has now passed the turning point of both curves and is on the right side of a positive U-shaped curve. Regarding coefficient values, direct residential energy consumption is more significantly influenced by the urbanization rate than indirect residential energy consumption. The relationship between indirect residential energy consumption and urbanization rate is more aligned with the positive linear correlation. The level of economic development most significantly influences the model estimation results.

3. The impact of urbanization on residential energy consumption is not the same in the three regions of East, Central, and West China. For direct residential energy consumption, the east, central and western regions are all on the right side of the positive U-shaped curve, i.e., the current residential energy consumption increases with the urbanization rate. Regarding indirect residential energy consumption, the development of urbanization has played a more prominent role in enhancing the central region, but the effect on the east and western regions is relatively small; for total residential energy consumption, the east, west, and other significant areas are also on the (5) The results of the study also show that urbanization has not played an essential role in the central region. In contrast, its position in the eastern part is weaker than in the western area. The study results also show the function of other control variables on residential energy consumption. Among them, the level of economic development is significantly positive at the 1% level of significance in all four regions of the model for direct, indirect, and complete residential energy consumption, indicating that this variable has a more significant impact. The level of economic development mainly influences indirect residential energy consumption. Population density hurts direct residential energy consumption, positively affecting indirect residential energy consumption. This may be because, with population agglomeration, the rational planning of mass transportation, such as metro and light rail, is conducive to saving intermediate costs in transportation and living production, bringing into play the spatial agglomeration effect and thus reducing residential direct energy consumption. However, at the same time, the agglomeration effect can also promote economic development and increase residents' income, thus increasing their indirect energy consumption. It is also worth noting that the impact of population accumulation is not the same in the eastern, central and western regions, indicating that moderate population agglomeration is conducive to reducing residential energy consumption. Nevertheless, excessive population agglomeration is not conducive to lowering residential energy consumption.

Supporting information

S1 Dataset.
(XLSX)

Author Contributions

Conceptualization: Weilong Wu, Youna lin.

Data curation: Weilong Wu, Youna lin.

Formal analysis: Youna lin.

Funding acquisition: Weilong Wu, Youna lin.

Investigation: Youna lin.

Methodology: Weilong Wu, Youna lin.

Project administration: Youna lin.

Resources: Youna lin.

Software: Weilong Wu, Youna lin.

Supervision: Youna lin.

Validation: Youna lin.

Visualization: Youna lin.

Writing – original draft: Weilong Wu, Youna lin.

Writing – review & editing: Youna lin.

References

1. Zheng W, Walsh PP. Economic growth, urbanization and energy consumption—A provincial level analysis of China. Energy Econ. 2019; 80: 449–462.
2. Wang S, Fang C, Guan X, Bo P, Ma H. Urbanisation, energy consumption, and carbon dioxide emissions in China: A panel data analysis of China's provinces. Appl Energy. 2014; 136: 738–749.
3. Zhou Z, Qin Q, France N. Epistemological dominance and ignorance of the comparative advantages of China's shale gas: Evidence from international scientific journals. Energy Policy. 2020; 138: 1126–1149.
4. Mol A. The Refinement of Production: Ecological Modernization Theory and the Chemical Industry. Environ Impact Assess Rev. 1996; 16: 57–61.
5. Buttel TH. Ecological modernization as social theory. Geoforum. 2000; 31: 57–65.
6. Baris K. The role of coal in energy policy and sustainable development of Turkey: Is it compatible to the EU energy policy? Energy Policy. 2011; 39: 1754–1763.
7. Stringer LC, Dougill AJ, Dyer JC, Vincent K, Fritzsche F, Leventon J, et al. Advancing climate compatible development: lessons from southern Africa. Reg Environ Chang. 2014; 14: 713–725.
8. Mol Arthur PJ. Ecological Modernization and the Global Economy. Glob Environ Polit. 2006; 2: 92–115.
9. Krishnamurthy C, KristrM B. Determinants of the Price-Premium for Green Energy: Evidence from an OECD Cross-Section. Environ Resour Econ. 2016; 64: 173–204.
10. Smith VL, Font X. Volunteer tourism, greenwashing and understanding responsible marketing using market signalling theory. J Sustain Tour. 2014; 22: 942–963.
11. Adua L, York R, Schuelke-Leech BA. The human dimensions of climate change: A micro-level assessment of views from the ecological modernization, political economy and human ecology perspectives. Soc Sci Res. 2016; 56: 26–43. https://doi.org/10.1016/j.ssresearch.2015.10.003 PMID: 26857170
12. Duru M, Theron D O. Livestock system sustainability and resilience in intensive production zones: which form of ecological modernization? Reg Environ Chang. 2015; 15: 1651–1665.

13. Christoff P. Ecological modernisation, ecological modernities. Env Polit. 2007; 5: 476–500.

14. Lynn Kevin. A triple "S" for sustainability: Credit ratings agencies and their influence on the ecological modernization of an electricity utility. Util Policy. 2013; 27: 9–14.

15. Musakwa W, Niekerk A V. Implications of land use change for the sustainability of urban areas: A case study of Stellenbosch, South Africa. Cities. 2013; 32: 143–156.

16. Matthias F, Maximilian F, Christoph H, Fred K. "Festivalisation" of Urban Governance in South African Cities: Framing the Urban Social Sustainability of Mega-Event Driven Development from Below. Sustainability. 2013; 5: 5225–5248.

17. Mclennan CL, Pham TD, Ruhanen L, Ritchie BW, Moyle B. Counter-factual scenario planning for long-range sustainable local-level tourism transformation. J Sustain Tour. 2012; 20: 801–822.

18. Czamanski D, Roth R. Characteristic time, developers' behavior and leapfrogging dynamics of high-rise buildings. Ann Reg Sci. 2011; 46: 101–118.

19. Keisuke S, Akiko I, Makoto Y. Spatial patterns of population turnover in a Japanese Regional City for urban regeneration against population decline: Is Compact City policy effective? Cities. 2018; 81: 230–241.

20. Lee S, Lee B. The influence of urban form on GHG emissions in the U.S. household sector. Energy Policy. 2014; 68: 534–549.

21. Shammin MR, Herendeen RA, Hanson MJ, Wilson EJH. A multivariate analysis of the energy intensity of sprawl versus compact living in the U.S. for 2003. Ecol Econ. 2010; 69: 2363–2373.

22. Shim GE, Rhee SM, Ahn KH, Chung SB. The relationship between the characteristics of transportation energy consumption and urban form. Ann Reg Sci. 2006; 40: 351–367.

23. Jones DW. How urbanization affects energy-use in developing countries. Energy Policy. 2007; 19: 621–630.

24. Parikh J, Shukla V. Urbanization, energy use and greenhouse effects in economic development. Results from a cross-national study of developing countries. Glob Environ Chang. 1995; 5: 87–103.

25. Jiang Z, Lin B. China's energy demand and its characteristics in the industrialization and urbanization process. Energy Policy. 2013; 60: 583–585.

26. Fang C, Liu H, Li G, Sun D, Zhuang M. Estimating the Impact of Urbanization on Air Quality in China Using Spatial Regression Models. Sustainability. 2015; 2015: 15570–15592.

27. Yu Y, Zhang N, Kim JD. Impact of urbanization on energy demand: An empirical study of the Yangtze River Economic Belt in China. Energy Policy. 2020; 139: 122–138.

28. Smith CJ. Social geography of sexually transmitted diseases in China: Exploring the role of migration and urbanisation. Asia Pac Viewp. 2005; 46: 65–80.

29. Solarin SA, Shahbaz M. Trivariate causality between economic growth, urbanisation and electricity consumption in Angola: Cointegration and causality analysis. Energy Policy. 2013; 60: 876–884.

30. Alam MJ, Begum IA, Buysse J, Huylenbroeck GV. Energy consumption, carbon emissions and economic growth nexus in Bangladesh: Cointegration and dynamic causality analysis. Energy Policy. 2012; 45: p.217–225.

31. Shahbaz M, Lean HH. Does financial development increase energy consumption? The role of industrialization and urbanization in Tunisia. Energy Policy. 2012; 40: 473–479.

32. Yang J, Wang Y, Xue B, Li Y, Xiao X, (Cecilia) Xia J, et al. Contribution of urban ventilation to the thermal environment and urban energy demand: Different climate background perspectives. Sci Total Environ. 2021; 795: 148791. https://doi.org/10.1016/j.scitotenv.2021.148791 PMID: 34237531

33. Yang J, Zhang W, Zhang Z. Impacts of urbanization on renewable energy consumption in China. J Clean Prod. 2016; 114: 443–451.

34. He BJ, Li Y, Miao Y, Mou B, Zhou Y. Overview of rural building energy efficiency in China. Energy Policy. 2014; 69: 385–396.

35. He BJ, Yang L, Ye M. Building energy efficiency in China rural areas: Situation, drawbacks, challenges, corresponding measures and policies. Sustain Cities Soc. 2014; 26: 449–465.

36. Pucher J. CITIES AND AUTOMOBILE DEPENDENCE: AN INTERNATIONAL SOURCEBOOK. Gower Tech. 1989; 24: 222–238.

37. Lafrance IL. Modelling the electricity consumption of cities: effect of urban density. Energy Econ. 1999; 26: 49–55.

38. Pachauri S, Jiang L. The household energy transition in India and China. Energy Policy. 2008; 36: 4022–4035.

39. Ke L, Lin B. Impacts of urbanization and industrialization on energy consumption/CO 2 emissions: Does the level of development matter? Renew Sustain Energy Rev. 2015; 52: 1107–1122.

40. Mishra V, Sharma S, Smyth R. Are fluctuations in energy consumption per capita transitory? Evidence from a panel of Pacific Island countries. Energy Policy. 2009; 37: 2318–2326.

41. Delbosc A, Kroesen M, Wee B V, Haas MD. Linear, non-linear, bi-directional? Testing the nature of the relationship between mobility and satisfaction with life. Transportation (Amst). 2019; 14: 526–553.

42. D DF., B IB, A DS, A ASP, C GK. On the way towards smart energy supply in cities: The impact of interconnecting geographically distributed district heating grids on the energy system. Energy. 2017; 137: 941–960.

43. Essah EA, Ofetotse EL. Energy supply, consumption and access dynamics in Botswana. Sustain Cities Soc. 2014; 12: 76–84.

44. Mcmillan TE. The relative influence of urban form on a child's travel mode to school. Transp Res Part A Policy Pract. 2007; 41: 69–79.

45. Purnell JQ, Katz ML, Andersen BL, Palesh O, Figueroa-Moseley C, Jean-Pierre P, et al. Social and cultural factors are related to perceived colorectal cancer screening benefits and intentions in African Americans. J Behav Med. 2010; 33: 24–34. https://doi.org/10.1007/s10865-009-9231-6 PMID: 19876727

46. Sadorsky P. Do urbanization and industrialization affect energy intensity in developing countries? Energy Econ. 2013; 37: 52–59.

47. Cairnes Lorraine. The Compact City: A Sustainable Urban Form. URBAN Des Int. 1996; 1: 293–294.
